# Unraveling the Mechanisms of Lithium‐Alloy Plating in Ag–C Anode: In situ SEM Study

**DOI:** 10.1002/advs.202404840

**Published:** 2025-02-08

**Authors:** Y. Kamikawa

**Affiliations:** ^1^ Research Division Nissan Motor Co., Ltd. Natsushima 1 Yokosuka Kanagawa 237‐0061 Japan; ^2^ Research Center for All‐Solid‐State Battery, Institute of Integrated Research Institute of Science Tokyo 4259 Nagatsuta, Midori‐ku Yokohama 226–8502 Japan

**Keywords:** Ag–C composite anode, first‐principles simulation, in situ SEM, operando XRD, STEM–EELS

## Abstract

The Ag–C composite anodes facilitate stable Li*_x_* Ag deposition in solid‐state batteries. However, the role of carbon and the kinetics of lithium migration and deposition in the composite structure remain unclear. Few studies have focused on this critical research area owing to a shortage of effective, non‐destructive characterization methods that can directly observe the Li alloy deposition process in solid‐state batteries in real time. In this study, Li alloy formation on the Ag–C anode is investigated through operando X‐ray diffraction (XRD) analysis and scanning electron microscopy combined with in situ probing. This enables the observation of the composition and morphology of the Li*_x_* Ag alloy as it evolves within the Ag–C composite anode during discharge. Further insights from scanning transmission electron microscopy–electron energy loss spectroscopy and first‐principles simulations on lithiophilicity and the energy barrier for lithium migration reveal a complex lithium migration and deposition mechanism within the Ag–C anode.

## Graphical abstract

Operando XRD, in situ SEM, and first‐principles simulations uncover the complex mechanism underlying the continuous and stable deposition in the Ag–C anode and complex interplay of the selective diffusion and deposition enabled by a nuanced balance of diffusivity and Li adsorption energy among constituent nanoparticles.

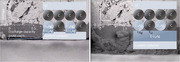

## 1 Introduction

Li metal is a highly promising anode material for application in high‐energy‐density rechargeable batteries owing to its high theoretical specific capacity (3860 mAh g^−1^).^[^ ^1^, ^2^ ^]^ However, inhomogeneous dendrite growth on Li metal during battery cycling leads to short circuits, which limits its use as an anode material.^[^ ^3^, ^4^ ^]^ Applications of different strategies based on artificial solid electrolyte layers have successfully mitigated the detrimental growth of Li dendrites by alleviating the generation of the space charge area and heterogeneous nucleation on the anode surface.^[^ ^5^, ^6^ ^]^ In this context, the presence of lithiophilic sites^[^ ^7^, ^8^ ^]^ on the stainless steel (SUS) current collector reduces the overpotential^[^ ^9^ ^]^ and maintains the stability of Li metal during discharging and charging.^[^ ^10^ ^]^ In light of the crucial role of nanoscale nucleation sites in controlling the Li plating and stripping behavior of Li metal anodes, metal nanoparticles (NPs) with low overpotentials for Li nucleation, such as Ag, Zn, Au, and Mg, have been investigated for their abilities to preferentially nucleate, dissolve into Li metal, and react with Li to form multiple alloy phases. This can reduce the nucleation barrier and guide Li deposition within carbonaceous host materials.^[^ ^11^, ^12^, ^13^, ^14^ ^]^ Nano‐Ag has been used as an anode in Li‐ion batteries for over a decade; however, its electrochemical performance is sensitive to the applied current densities.^[^ ^15^, ^16^ ^]^ Notably, these alloy‐forming Ag nanoseeds combined with carbon shells^[^ ^8^ ^]^ and carbon macroporous fibers^[^ ^11^ ^]^ facilitate stable Li deposition and superior Coulombic efficiency.

These nanoseeds can regulate the Li plating kinetics to prevent dendrite formation by forming an ordered Li*_x_* Ag alloy layer beneath the carbon layer rather than disordered lithiation on the Ag–C composite anode.^[^ ^17^ ^]^ Despite the superior performance of the Ag–C composite anode to that of conventional Li*_x_* Ag alloy anodes, the role of carbon and the kinetics of Li migration and deposition in the composite structure remain unclear. Moreover, effective, non‐destructive characterization methods for the operando observation of the Li deposition process in solid‐state batteries have not been reported thus far.

This study revealed the Li alloy formation in an Ag–C|Li_6_PS_5_Cl(LPSCl)|Li solid‐state battery for the first time through operando X‐ray diffraction (XRD) analysis and scanning electron microscopy (SEM) equipped with an in situ probing station. Specifically, these techniques were used to monitor the evolution of the Li–Ag alloy morphology and the Li–Ag composition in the Ag–C composite anode during discharge. In conjunction with insights obtained from scanning transmission electron microscopy–electron energy loss spectroscopy (STEM–EELS) and first‐principles calculations on lithiophilicity and the energy barrier for Li migration, the in situ observation results elucidate the critical role of selective deposition and diffusion, facilitated by a nuanced balance between the adsorption energy and Li mobility among the constituent NPs, which enables the continuous and ordered deposition of δ‐LiAg.

## 2 Results and Discussion

**Figure 1:**
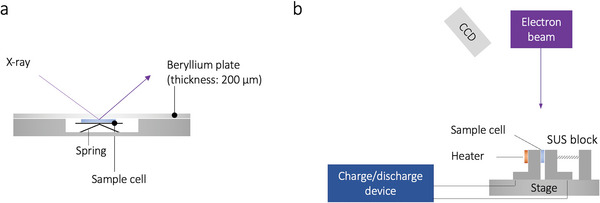
Schematics of a) operando XRD analysis and b) in situ SEM.

**Figure 2:**
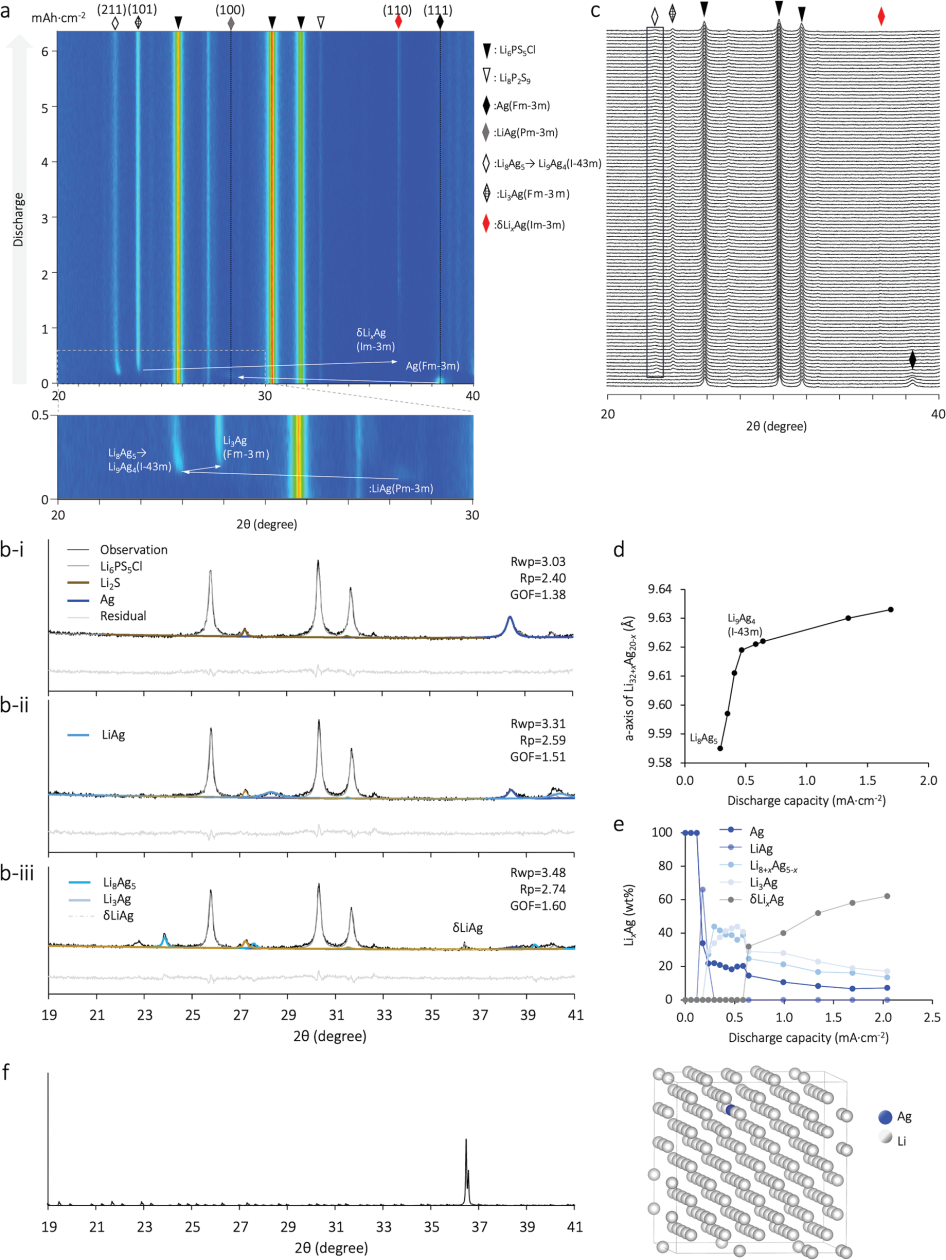
a) Real‐time phase tracking of the Ag–C anode at different potential stages in the half cell with reference to the Li foil, with a magnified view (bottom) of the current‐density range 0–0.5 mAh cm^−2^, where in the most phase change occur; b) peak‐fitting results at a discharge capacity of i) 0 mAh cm^−2^, ii) 0.17 mAh cm^−2^, and iii) 2.04 mAh cm^−2^; c) XRD pattern of the Ag–C electrode during the operando XRD test, where the black rectangle indicates the broadening of the Li_9_Ag_4_ peak; d) *a*‐axis constant of Li_32+_ *_x_* Ag_20‐_ *_x_*; e) dependence of the Li*_x_* Ag composition on the stage of charge; and f) XRD pattern and atomic structure of a δ‐phase Li*_x_* Ag solid solution, where blue and gray spheres indicate Ag and Li atoms, respectively.

An operando XRD analysis was conducted to confirm the compositional evolution of the Li*_x_* Ag alloy within the Ag–C anode (**Figure** **1**a). Before discharging, the Ag peaks are observed at 38.2° (**Figure** **2**a,b‐i). A discharge of 0.17 mA cm^−2^ during lithiation led to the gradual appearance of the β(Li–Ag) phase (Figure 2b‐ii), which was then replaced by γ_3_(Li_8_Ag_5_). The larger lattice constant (9.59 Å) than the theoretical value (9.47 Å) could be attributed to the coexistence of other forms of Li*_x_* Ag, as Li_8_Ag_5_ does not exist in a perfectly ordered form.^[^ ^18^ ^]^ Moreover, γ_3_(Li_8_Ag_5_) abruptly transformed into γ_3_(Li_9_Ag_4_). The lattice constant of Li_9_Ag_4_ was 9.62 Å, which is consistent with previous XRD results for single‐crystal Li_9_Ag_4_.^[^ ^19^ ^]^ The replacement of Li_8_Ag_5_ with Li_9_Ag_4_, which has a disordered structure, is also evidenced by the broadened peak at 22.9° at 0.41 mA cm^−2^ (Figure 2c). The γ_3_(Li_3_Ag) phase coexisted with Li_9_Ag_4_ as the discharge proceeded to 0.41 mA cm^−2^ (Figure 2a). Further lithiation negatively shifted the γ_3_(Li_9_Ag_4_) peak (Figure 2a) with continued Li dosing in the solid solution,^[^ ^16^ ^]^ leading to the expansion of the γ_3_ phase (Figure 2d).  Further discharge above 0.52 mAh cm^−2^ resulted in the formation of a Li‐rich solid solution (Figure 2b‐iii, 2e). Figure 2f shows the first‐principles XRD pattern results of the δ‐phase Li*_x_* Ag, in which a single Li atom in a 5 × 5 × 5 supercell of Li with a body‐centered cubic structure is replaced by an Ag atom. The simulated XRD patterns of the δ‐phase Li*_x_* Ag solid solution exhibit a peak at 36.4°, which is identical to that for metallic Li,^[^ ^20^ ^]^ corresponding to the (110) plane. To confirm the elemental distribution of this deposition, an in situ SEM experiment was conducted.

**Figure 3:**
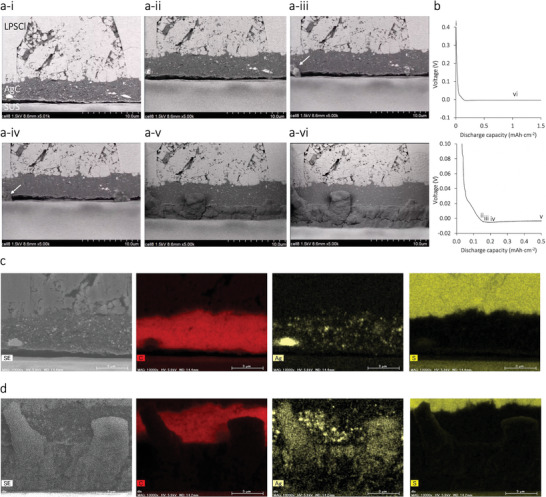
a) Real‐time in situ SEM observations during the b) discharge of the Ag–C anode. c,d) SEM–EDX results for the Ag–C anode c) before discharge and d) at a discharge capacity of 1.5 mAh cm^−2^. (See Movie S1 for the full reaction).

In situ SEM measurements were performed to confirm the morphology of the deposited Li–Ag alloy at different discharge capacities (Figure 1b; a video showing the dynamics of the Li*_x_* Ag morphology observed via in situ SEM is presented in Movie S1). Lithiation at the Ag–C anode was observed in the symmetric Ag–C|LPSCl|Li cell. Li*_x_* Ag alloy plating was controlled by discharging solid‐state batteries to different capacities. Lithiation was first observed within the Ag–C layer until the discharge capacity reached 0.18 mAh cm^−2^ (**Figure** **3**a,b). No significant deformation of the Ag–C NP alignment or detachment of the interface between the Ag–C anode and solid electrolyte was observed, indicating that the seeds for Li plating homogenized the volume expansion during lithiation. At a capacity of >0.18 mAh cm^−2^, extra Li was deposited on the surface of Li_x_Ag particles (white arrow in Figure 3a‐iii).

The operando XRD results indicate the copresence of Li_3_Ag and Li_9_Ag_4_ throughout the process of δ‐LiAg formation during discharge. Considering the phase transition of Li*_x_* Ag, during which the formation of Li_9_Ag_4_ is followed by a transition to Li_3_Ag and δ‐LiAg,^[^ ^10^, ^16^ ^]^ Li_3_Ag is the Li_*x*_Ag composition of the deposition site for δ‐LiAg. Upon further lithiation, the deposition layer was uniformly formed at the Ag–C/SUS interface (Figure 3a‐v). SEM–energy dispersive X‐ray spectroscopy (EDX) analysis (Figure 3d) was used to investigate the distribution of Ag species throughout the deposition phase. This elemental mapping, obtained through in situ SEM and a first‐principles simulation of the XRD pattern of δ‐LiAg, confirms that the XRD peak at 36.4° observed through operando XRD is attributable to δ‐LiAg. In contrast to the dynamics of δ‐LiAg morphology, Li*_x_* Ag (*x* < 3) and carbon black (CB) maintained their original composite NP structure throughout the discharge process (Figure 3a,d). The δ‐LiAg content increased with an incremental reduction in the contents of Li_3_Ag and Li_9_Ag_4_ (Figure 2e); however, Li_3_Ag and Li_9_Ag_4_ evidently persisted even when the discharge capacity reached 6.2 mAh cm^−2^. Furthermore, the CB distribution remained unchanged throughout the discharge process (Figure 3c,d).

**Figure 4:**
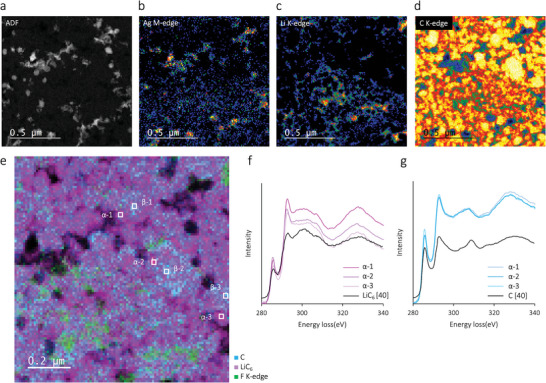
STEM‐EELS of the Ag–C anode: a) ADF TEM image and corresponding EELS chemical composition maps for the b) Ag M‐edge, c) Li K‐edge, and d) C K‐edge. e) Multiple linear least‐squares fitting results for C and LiC_6_ based on C K‐edge spectrum. The blue and purple domains represent C and LiC_6_, respectively, obtained through carbon K‐edge fitting, while the green domains correspond to the mapping of F K‐edge, corresponding to PVDF. f,g) K‐edge spectrua from the f) edge and g) center of the CB particles.

To elucidate the lithiation in the nanocomposite structure of Li*_x_* Ag NPs and CB particles, STEM–EELS was conducted for the Ag–C|LPSCl|Li cell sample at a discharge capacity of 1.55 mAh cm^−2^. The bright part of the annular dark‐field (ADF) image (**Figure** **4**a) and the elemental mapping of Ag (Figure 4b) as well as comparison with those of Li (Figure 4c) and C (Figure 4d) confirm that Li species predominantly exists as the Li*_x_* Ag alloy, whereas Li is scarce in the domain of the C species. To further investigate the lithiation involving CB, the distribution of LiC_6_ was analyzed. Figure 4e shows the multiple linear least‐squares fitting results for C and LiC_6_ based on the C K‐edge spectrum. The green‐highlighted domain in Figure 4e shows the F K‐edge mapping, corresponding to the polyvinylidene fluoride (PVDF) that combines the Ag NPs and CB particles. Evidently, CB lithiation occurs only at the CB surface (Figure 4d), whereas the entire cross‐section of the Ag NPs is lithiated (Figure 4b,c). This spatial separation of LiC_6_ and C is further confirmed by the C K‐edge spectrum for the points at the edge (Figure 4f) and center (Figure 4g) of the CB NPs.

**Figure 5:**
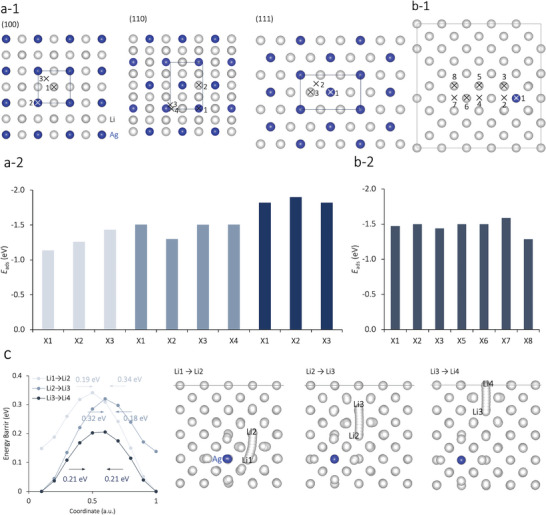
Li adsorption energy (*E*_ads_) for a) Li_3_Ag and b) δ‐LiAg; c) energy barrier for three different Li migration paths on the δ‐LiAg surface at different distances from the Ag atom.

Despite significant previous efforts, lithiophilicity and Li mobility in Li*_x_* Ag have yet to be comprehensively understood. However, recent first‐principles calculations have partly demonstrated lithiophilicity and Li mobility of a Li*_x_* Ag alloy at certain stages of lithiation. These calculations indicated a higher energy barrier for Li migration in Li_3_Ag^[^ ^21^ ^]^ than in LiC_6_,^[^ ^22^ ^]^ as well as the superior lithiophilicity of Li*_x_* Ag (*x* = 1.0) compared to that of the LiC_6_ surface.^[^ ^23^ ^]^ Considering the nanoscale distribution of lithiated species, as well as the balance of the lithiophilicity and energy barrier for Li migration in Li*_x_* Ag and Li*_y_* C, the influence of CB in the Ag–C composite anode is attributable to the provision of rapid Li diffusion pathways for Ag NPs, enabling continuous and stable lithiation in these Ag NPs. This explains the poor rate performance of Ag anodes in the absence of CB.^[^ ^15^ ^]^ To further investigate the lithiophilicity underlying the selective deposition of δ‐LiAg on the Li_3_Ag surface rather than the LiC_6_, as observed through in situ SEM and STEM−EELS results, and to determine the origin of the homogeneous distribution of Ag atoms in δ‐LiAg, the lithiophilicity and energy barrier for Li migration were evaluated through first‐principles simulations. The results indicated that δ‐LiAg has an adsorption energy (*E*_ads_) as low as that of Li_3_Ag (**Figure** **5**a), regardless of the distance from the Ag atom (Figure 5b). The adsorption energy of both forms of Li*_x_* Ag is much lower than that of LiC_6_ (*E*_ads_ = −0.12 eV).^[^ ^23^ ^]^ This explains the stable selective deposition of δ‐LiAg on the Li_3_Ag surface rather than on the LiC_6_ surface, as evidenced by in situ SEM and STEM–EELS results. A nudged elastic band (NEB) simulation was performed in which the migration paths were varied on the δ‐LiAg surface, and the results indicated that δ‐LiAg has a lower energy barrier for Li migration (*E*_b_ = 0.19−0.34 eV) than Li_3_Ag (*E*_b_ = 0.76 eV).^[^ ^22^ ^]^ This lower *E*_b_ value of δ‐LiAg explains the homogeneously distributed Ag atoms in δ‐LiAg observed via SEM–EDX (Figure 3d). Notably, the persistently lower values of *E*_b_ and *E*_ads_, even when the distance between the migration path (Figure 5c) or adsorption site from the Ag atom is over twice the Li–Li unit distance, confirm the ability of δ‐LiAg to continuously dissolve Li atoms by homogenizing the Ag distribution, even at low Ag concentrations in δ‐LiAg (a single Ag atom in the 5 × 5 × 5 supercell of Li).

Results from operando XRD, in situ SEM, and first‐principles simulations reveal the complex mechanism underlying the continuous and stable deposition of Li*_x_* Ag alloy in the Ag–C anode. This involves a complex interplay of selective diffusion and deposition facilitated by a nuanced balance of diffusivity and Li adsorption energy among constituent NPs. In the conventional Li metal anode, the poor mobility of Li and vacancies^[^ ^24^, ^25^ ^]^ leads to heterogeneous Li deposition at the Li/solid electrolyte interface. This heterogeneity is further enhanced by the inhomogeneous electric potential distribution induced by interfacial voids at the Li/solid electrolyte interface.^[^ ^26^, ^27^ ^]^

**Figure 6:**
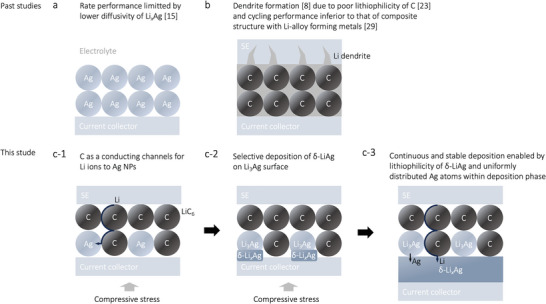
Comparison of the deposition mechanism in the a) Ag anode, b) C interlayer, and c) Ag–C nanocomposite anode.

Despite the lower energy barrier of Li_3_Ag compared to that of the Li(100) surface (*E*_b_ = 0.48 eV^[^ ^28^ ^]^), the Ag anode in the absence of CB has been reported to exhibit poor cycling performance owing to its limited diffusivity (**Figure** **6**a).^[^ ^15^ ^]^ The application of a CB interfacial layer in the absence of alloy‐forming metals affords conducting channels for Li ions. However, the CB interfacial layer exhibits low cycling performance in the absence of Li‐alloy‐forming metals.^[^ ^29^ ^]^ Such anode structures lead to intensive Li dendrite growth (Figure 6b),^[^ ^8^ ^]^ which is possibly attributed to the poor lithiophilicity of LiC_6_^[^ ^23^ ^]^ and the consequential high overpotential (−24 mV) for Li deposition.^[^ ^8^ ^]^

In contrast, upon using the Ag–C nanocomposite structure, the superior mobility of Li in graphite (*E*_b_ = 0.08–0.24 eV^[^ ^30^ ^]^) and LiC_6_ (*E*_b_ = 0.22 eV^[^ ^31^ ^]^) facilitate the supply of Li to the Ag NP surface throughout the anode structure (Figure 6c‐1). The higher lithiophilicity of Li*_x_* Ag, compared to that of LiC_6_, promotes Li adsorption, resulting in selective lithiation in Ag NPs, whereas the CB particles undergo lithiation only at the surface.

The formation of Li_3_Ag is followed by the deposition of δ‐LiAg upon further lithiation. Notably, the in situ SEM results show that δ‐LiAg is selectively deposited on the Li_3_Ag surface, rather than from the entire Ag–C/SUS interface (Figure 6c‐2). As evident from the first‐principles simulation results, this selective deposition is attributable to the superior lithiophilicity of Li_3_Ag and δ‐LiAg in comparison with that of LiC_6_ (Figure 5a‐2,b‐2). To confirm the energetic feasibility of Ag atom diffusion at the Li_3_Ag(100)/Li(100) interface, the atom‐exchange properties at the Li_3_Ag/Li(100) interface with a single Li vacancy in Li metal were analyzed (Figure S1, Supporting Information). The structural relaxation results confirm the favorable dissolution of Ag atoms from Li_3_Ag into the Li metal phase (Figure S1, Supporting Information).

Upon further lithiation, δ‐LiAg deposits from the Li_3_Ag surface, suggesting the critical role of Li_3_Ag as a nanoseed for δ‐LiAg deposition. The rest of the discharge process is enabled by the continuous deposition of δ‐LiAg over the entire Ag–C/SUS interface to compensate for the incremental decrease in the Li_3_Ag and Li_9_Ag_4_ contents, suggesting the continuous dissolution of Ag atoms into the deposited phase (Figure 6c‐3).

In contrast to the dynamics of the δ‐LiAg morphology, Li*_x_* Ag NPs persist throughout the discharge process, thus maintaining the original nanocomposite structure of the Ag NPs and CB particles. Consequently, further lithiation leads to the formation of an ordered bi‐layered structure comprising an Ag–C NP layer and δ‐LiAg layer. The complex mechanism underlying the stable lithiation in the Ag–C anode involves the interplay of Li diffusion and deposition enabled by the nuanced balance of the Li mobility and lithiophilicity of each constituent in the composite anode. These dynamics during the lithiation process provide an avenue for designing Li‐alloy‐forming anode materials.

Moreover, the cell thickness and its variation depending on the stage of lithiation are essential for designing the Ag–C anode cell and its compression structure within the battery module. The evolutionary process of the Li*_x_* Ag composition and morphology revealed in this study provides an essential baseline for designing Ag–C anodes by informing the alignment of Li*_x_* Ag and Li*_x_* C during each stage of lithiation. Further, considerable efforts have been directed toward improving Li‐alloy‐forming anodes.^[^ ^32^, ^33^ ^]^ The current research on Li metal cells predominantly focused on the use of Li metal as the anode.^[^ ^34^ ^]^ As lithium oxide cathodes are pre‐lithiated, surplus Li decreases the energy density relative to the theoretical limit.^[^ ^35^ ^]^ Solid‐state batteries based on the anode‐free concept have been heralded as the next generation of energy storage solutions because they eliminate excess Li, increasing the energy density.^[^ ^36^ ^]^ Alloy‐forming anodes have been extensively explored in the research on anodes free of precious metals.^[^ ^37^ ^]^ Li–Mg alloys are candidates as alternatives to Li–Ag alloys; however, their cycling performance is limited because of poor diffusivity.^[^ ^38^ ^]^ This difficulty is attributed to the inherent tradeoff between adsorption energy and diffusion energy barriers in alloy‐forming metals, as highlighted by previous computational screening studies.^[^ ^39^ ^]^ A combinatorial approach that involves designing the lithiophilicity and Li mobility of the Li‐alloy‐forming metal and optimizing the composite structure to facilitate the migration of Li onto the Li‐alloy‐forming metal surface could serve as a strategy for developing novel Li‐alloy‐forming anodes.

## 3 Conclusion 

In this study, Li*_x_* Ag alloy formation was observed for the first time in an Ag–C|LPSCl|Li solid‐state battery by employing an in situ probing station during SEM and operando XRD. The evolution of the morphology and composition of Li*_x_* Ag in the Ag–C composite anode is observed at the interface between the solid electrolyte and the SUS current collector. The results showed the formation of Li*_x_* Ag (*x* ≤ 3) within the Ag–C composite layer, followed by the continuous deposition of δ‐LiAg at the Li_3_Ag/SUS current collector interface during the lithiation process. Combined with insights from STEM–EELS and first‐principles simulations on lithiophilicity and the energy barrier for Li migration, the in situ observation results revealed the critical role of selective deposition and diffusion enabled by a nuanced balance between the adsorption energy and Li mobility among the constituent NPs, which allow the continuous and ordered deposition of δ‐LiAg. The intricate underlying mechanism elucidated in this study could inform the designer of an Ag–C nanocomposite anode structure that optimizes the functional balance of each constituent and ensures continuous and orderly lithiation, while minimizing the use of each type of NPs. Furthermore, the critical role of this balance among the lithiophilicity and diffusivity of each constituent in the Ag–C composite anode revealed in this study could open a new avenue toward the development of alternative alloy‐based anodes by enhancing and tailoring these properties within the composite structure, addressing the inherent limitations of conventional methods that rely on a single alloy‐forming metal.

## 4 Experimental and First‐Principles Simulations

### Sample Preparation

Ag NPs (D50 = 60 nm) and CB particles (D50 = 35 nm) were mixed in a weight ratio of 1:3 in N‐methylpyrrolidone, with 7 wt.% PVDF. The slurry was then coated on 10 µm‐thick SUS foil and dried under vacuum at 100 °C for >10 h. The thickness of the Ag–C nanocomposite layer was 10 µm. Li_6_PS_5_Cl powder (100 mg; Ampcera) was pressed into a pellet with a diameter of 10 mm at 400 MPa for 3 min. Then, the Ag–C anode (*∅* = 10 mm) was pressed onto the solid‐state battery pellet at 400 MPa to create the Ag–C/LPSCl interface. A Li metal foil (Honjo metal; *∅* = 9 mm, thickness = 150 µm) was placed on the opposite side of the LPSCl layer and compressed at 3 MPa to form the LPSCl/Li interface.

### Operando XRD

The operando XRD cell was made from an in situ holder with a 32 mm hole on the top casing, where a 0.2 mm‐thick beryllium disk was glued onto the X‐ray window (Figure 1a). A current density of 137 µA cm^−2^ (108 µA) and a plating time of 45 h were adopted to discharge the Ag–C anode. Operando XRD patterns of the Ag–C electrodes were collected every 26 min. The measurements were performed on a Bruker D8 ADVANCE instrument (Bruker Corp.) with Cu‐κα radiation in a 2θ range of 10°–60° at a step size of 0.01711° and 0.5 s step^−1^. The aluminum diffraction peak was used as the reference to correct the sample displacements. The crystallinity of Li*_x_* Ag was estimated using data from the Material Project and ICSD databases.

### In Situ SEM

The samples were attached to the cryostage of an Ar‐BIB milling system (IB‐19520, JEOL). Ar+BIB milling was performed to obtain cross‐sections of the samples for SEM observation. During the milling, the temperature of the cryostage was maintained at <–140 °C. Subsequently, in situ microscopic observations were conducted on the Ag–C anode during battery operation at 60 °C. In situ SEM studies were performed using an S‐4800 (Hitachi) equipped with an electrochemical testing instrument (XFlash, Bruker Inc.) under a discharge current of 0.108 mA and an acceleration voltage of 1.5 kV (Figure 1b).

### STEM–EELS

Cryo‐STEM characterization of these samples was performed through aberration‐corrected TEM using an ARM200F instrument (JEOL) equipped with a charge‐coupled device (CCD) camera (Gatan US4000) under an accelerating voltage of 200 kV. The beam size was set to be equivalent to the spatial step size. The microscope was equipped with a cold‐field‐emission electron gun (CFEG). A Gatan Quantum image filter (GIF Quantum ER, Gatan) was used to remove the inelastically scattered electrons. A standard Gatan side‐entry cryotransfer holder (DTCHVT, Gatan) was used to transfer samples to the microscope, and sample temperatures were maintained at ≈−170 °C during the experiment. Multiple linear least‐squares fitting was conducted to obtain the individual intensity map for C and LiC_6_ using the Gatan Microscopy Suite 3 software based on a reference spectrum from a previous report.^[^ ^40^ ^]^

### First‐Principles Simulations

Density functional theory (DFT) calculations were performed using Quantum ESPRESSO.^[^ ^41^ ^]^ The generalized gradient approximation (GGA) of Perdew−Burke−Ernzerhof (PBE) was used for the exchange‐correlation functional.^[^ ^42^ ^]^ Core–valence electron interaction was treated using projector‐augmented wave (PAW) pseudopotentials with a plane‐wave cutoff energy of 50 Ry and Gaussian smearing with a width of 0.05 eV. The convergence criteria for electronic self‐consistent iteration was set to 1 × 10^−7^ Ry. The Monkhorst–Pack k‐point mesh was selected based on the following rule: *k* × *a* ≥ 55 Å, where *a* is the length of the basis vector, and *k* is the number of k‐points in any direction. Following the prior first‐principles study on Li metal with a low content of the dopant element,^[^ ^38^ ^]^ the Li lattice along the plane is presumed to remain unchanged at a low Ag content. The Li(100) facet was selected because it has the lowest‐energy surface.^[^ ^43^, ^44^ ^]^ The migration activation energies were determined using the NEB method as implemented in Quantum ESPRESSO. Atoms were relaxed until the forces were <0.03 eV Å^−1^. Ten images were generated in the computation, separated by a spring constant of 5.0 eV Å^−2^. Structures of Li_3_Ag(100)/Li(100) interfaces were built using the lattice match algorithm^[^ ^45^ ^]^ by embedding several slabs in one cell having translation symmetry compatible with these slabs. The unit cells of the slabs were matched using a maximal lattice vector length mismatch of 0.03 Å and maximal angle mismatch of 0.01 rad. These interface models have a sandwich‐like structure because of periodic boundary conditions in the direction perpendicular to the interface.

## Conflict of Interest

The authors declare no conflict of interest.

## Supporting information



Supporting Information

Supplemental Movie 1

## Data Availability

The data that support the findings of this study are available from the corresponding author upon reasonable request.
