# Supplementary material for: Unraveling the Mechanisms of Lithium‐Alloy Plating in Ag–C Anode: In situ SEM Study
Source: Adv Sci (Weinh). 2025 Feb 8;12(13):2404840. doi: 10.1002/advs.202404840 (PMC11967751; doi:10.1002/advs.202404840)
Supplement: Supplementary file 1 — Supporting Information [file ADVS-12-2404840-s001.docx]

Supporting information

Unraveling the Mechanisms of Lithium-Alloy Plating in Ag–C anode: In-situ SEM Study

Y. Kamikawa*

*Research Division, Nissan Motor Co., Ltd. Natsushima 1, Yokosuka, Kanagawa, 237-0061*

*Research Center for All-Solid-State Battery, Institute of Integrated Research, Institute of Science Tokyo, 4259 Nagatsuta, Midori-ku, Yokohama 226-8502*

*Email: yu-kamikawa@mail.nissan.co.jp*


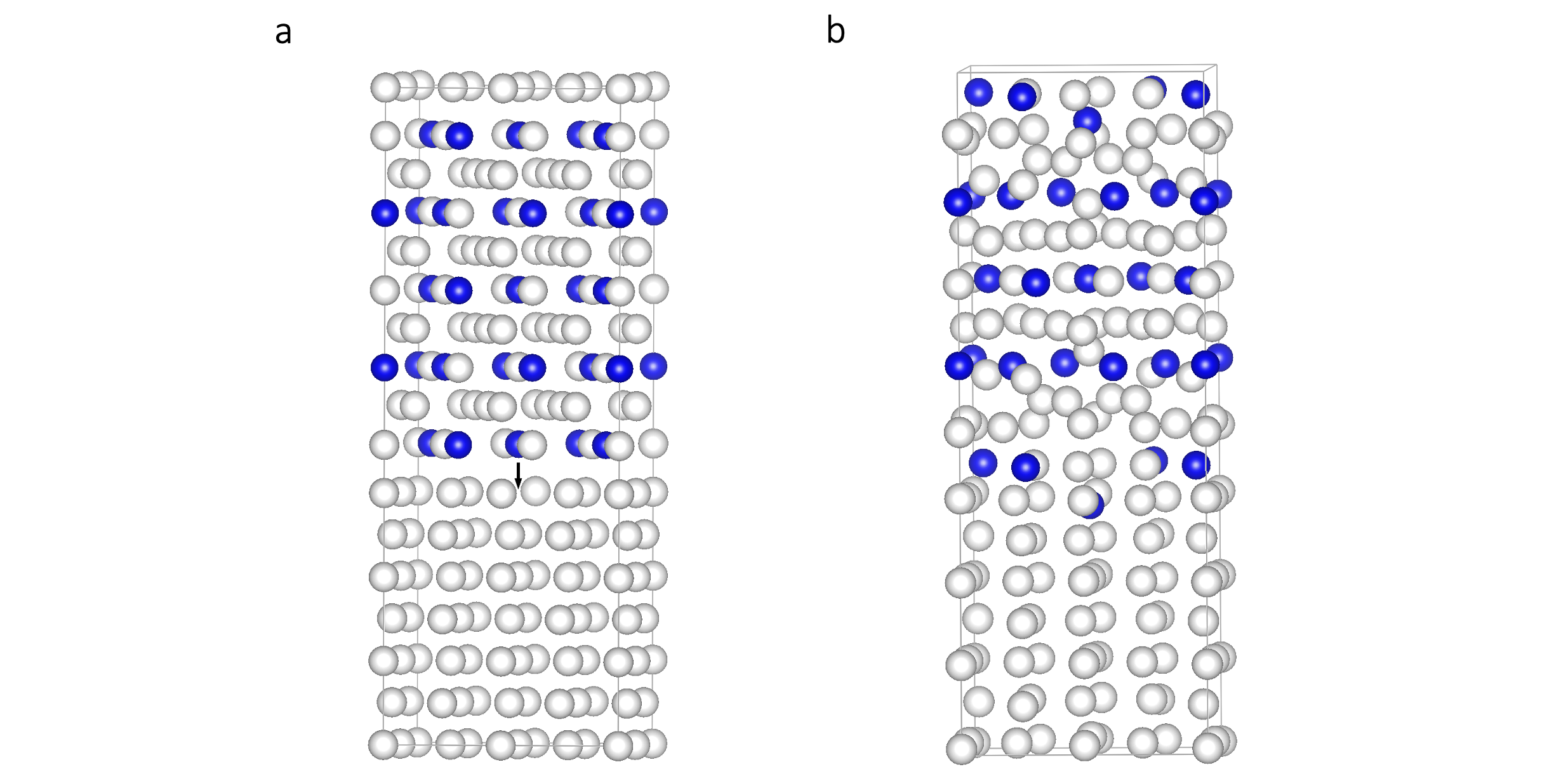


**Figure S1.** (a) Li_3_Ag(100)/Li(100) interfacial structure with a single Li vacancy in Li metal (black arrow). (b) Results of structural relaxation. Blue and gray spheres represent Ag and Li atoms, respectively.
